# Renewable juglone nanowires with size-dependent charge storage properties†

**DOI:** 10.1039/c7ra12489a

**Published:** 2018-01-09

**Authors:** Linlin Guo, Aifen Wang, Pengfei Hu, Aihua Tian, Rui Hao, Dandan Yu, Jie Yang, Dezhi Chen, Hua Wang

**Affiliations:** School of Chemistry, Beihang University Beijing 100191 PR China wanghua8651@buaa.edu.cn; School of Science, Hangzhou Dianzi University Hangzhou 310018 PR China; Key Laboratory of Jiangxi Province for Persistent Pollutants Control and Resources Recycle, Nanchang Hangkong University Nanchang 330063 PR China

## Abstract

Inspired by the biological metabolic process, some biomolecules with reversible redox functional groups have been used as promising electrode materials for rechargeable batteries, supercapacitors and other charge-storage devices. Although these biomolecule-based electrode materials possess remarkable beneficial properties, their controllable synthesis and morphology-related properties have been rarely studied. Herein, one dimensional nanostructures based on juglone biomolecules have been successfully fabricated by an antisolvent crystallization and self-assembly method. Moreover, the size effect on their electrochemical charge-storage properties has been investigated. It reveals that the diameters of the one dimensional nanostructure determine their electron/ion transport properties, and the juglone nanowires achieve a higher specific capacitance and rate capability. This work will promote the development of environmentally friendly and high-efficiency energy storage electrode materials.

## Abstract 2

Renewable juglone nanowires have been successfully fabricated, and their size effect on electrochemical charge-storage properties has been investigated.

## Graphical abstract

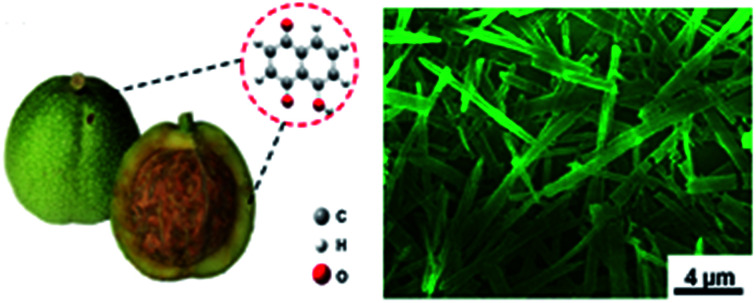

Currently, the development of high-performance electrochemical energy-storage materials and devices is attracting intensive interest. Conventional electrode materials involving transition metal compounds,^1–4^ elementary substances,^5–8^ and conductive polymers,^9–12^ with superior charge storage properties have been widely investigated. However, the poor biocompatibility, rising prices and depletion issues limit their sustainable applications due to their intrinsic material properties.^13^ Thus, exploring naturally abundant and renewable charge-storage materials with promising electrochemical performance is of great significance.

In the biological system, its metabolic process mainly relies on ions transport and energy exchanges of redox-active biomolecules with special functional groups such as carbonyl groups, carboxyl groups, and pteridine centres.^14^ Due to their abundance, sustainability, environmental benignity, these renewable and nature-derivable biomolecules with well-defined charge-storage behaviors are ideal alternatives to conventional electrode materials for the next-generation green energy-storage devices.^15–17^ For instance, biomolecules such as lignin,^18^ melanin,^19^ riboflavin,^20^ juglone^21^ and humic acid^22^ have been demonstrated as promising electrode materials for the rechargeable batteries, supercapacitors and other charge-storage devices. Although these biomolecule-based electrode materials possess remarkable beneficial properties, they are still confronted with several serious problems of poor conductivity and high electrochemical reaction impedance.^23^ For some conventional inorganic and organic active electrode materials, decreasing their size has been demonstrated to be effective strategies to enhance the electrochemical reaction kinetics by exposing more active sites to electrolytes and conductive agent.^8,24,25^ These results have strongly motivated us to develop biomolecule-based nanostructures, and investigated their size-correlative charge storage behavior.^26,27^

Herein, one dimensional (1D) nanostructures based on juglone, a renewable redox-active biomolecule which can be derived from matured fruits of black walnut and the green peel of juglandaceae, have been successfully fabricated by an antisolvent crystallization and self-assembly method.^28–30^ The size effect on the electrochemical charge-storage properties of these biomolecule-based 1D nanostructures have been investigated. It reveals that the electronic/ionic transport properties and charge-storage performance can be modulated by the size of self-assembled 1D nanostructures, and the samples with smaller diameter realize the higher specific capacitance and rate capability. Our work will provide insights for the development of high-performance biomolecule-based green energy-storage materials and devices.

Juglone, also called 5-hydroxy-1,4-naphthalenedione, is a nature-derivable biomolecule, and displays a well-defined redox behavior in acetonitrile due to its quinone groups (Fig. 1a and b).^31^ As organic molecule inherently, it is soluble in organic solvent and difficult to dissolve in water, so its nano-architectures could be condonably fabricated by an antisolvent crystallization strategy (Fig. 1c) and would carry out stably for charge storage in an aqueous electrolyte.^29,30^ Firstly, the juglone-biomolecule-based 1D nanostructures with different size were synthesized. The juglone micropillars with a mean diameter around 12 μm were prepared by directly recrystallizing a water/acetonitrile mixed solution of juglone at room temperature (Fig. 2a and d). Compared to commercially available raw juglone materials (Fig. S1†), the juglone micropillars could increase its charge storage performance, but its relative large size would still confine its contact with electrolyte, and thus remarkably reduce the reaction kinetics.^32^ To further improve the potential reaction kinetics, the juglone microwires with an average diameter about 1 μm (Fig. 2b and e) and juglone nanowires with a mean diameter about 550 nm (Fig. 2c and f) were fabricated at room temperature using the reprecipitation method.^33^ In the specific synthesis process, a high-concentration juglone acetonitrile solution is injected into water, which is a poor solvent for juglone molecules. Under stirring, juglone started to crystallize within a few seconds owing to its poor solubility in the water/acetonitrile mixture solvent and self-assembled into 1D nanostructures, and this procedure could be resulted from the π–π interaction.^29^ It can be found that the diameter of 1D juglone materials is adjustable by tuning the concentration of juglone in acetonitrile, and the higher concentration of juglone acetonitrile solution yields the smaller size of 1D juglone materials. The insert panels show the percentage of juglone micropillar, microwire, nanowire with different diameter coverage (Fig. 2d–f).

**Fig. 1:**
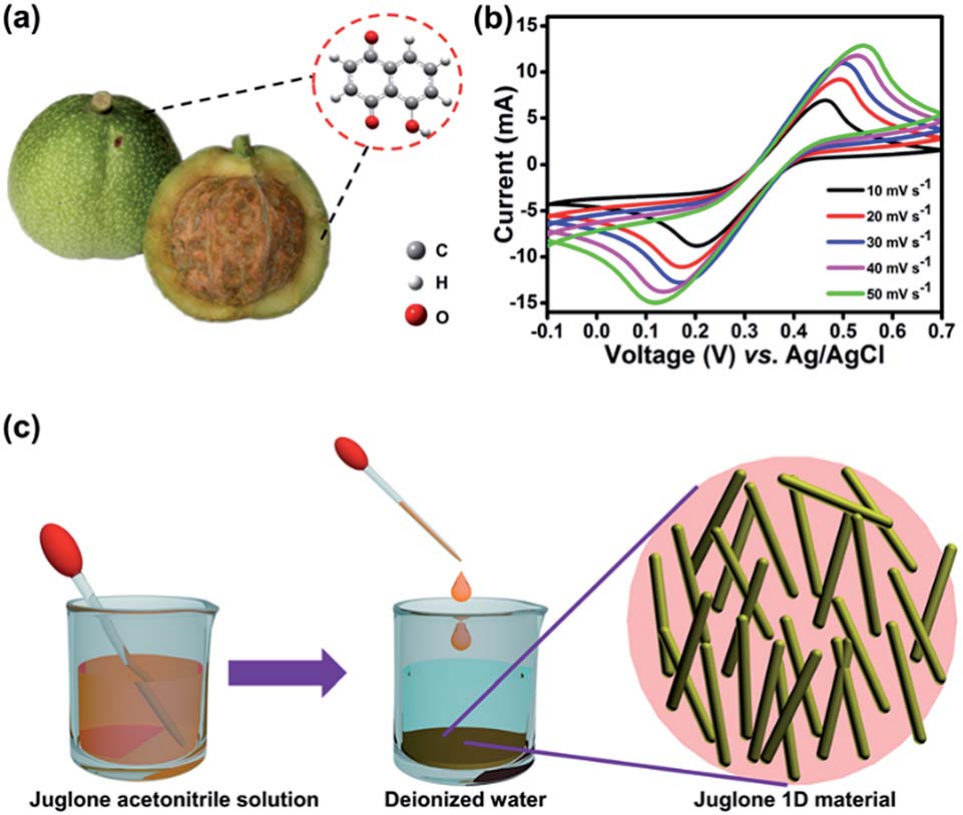
(a) Chemical structural formula of juglone biomolecules which can be derived from the bark of black walnuts. (b) Juglone redox activity verified in a mixed solution of acetonitrile/deionized water by a three-electrode system using Pt foils as both the counter and working electrodes, Ag/AgCl as the reference electrode, and 2.3 M H_2_SO_4_ as the electrolyte. (c) Schematically illustration of the fabrication of the juglone nanowire/microwire.

**Fig. 2:**
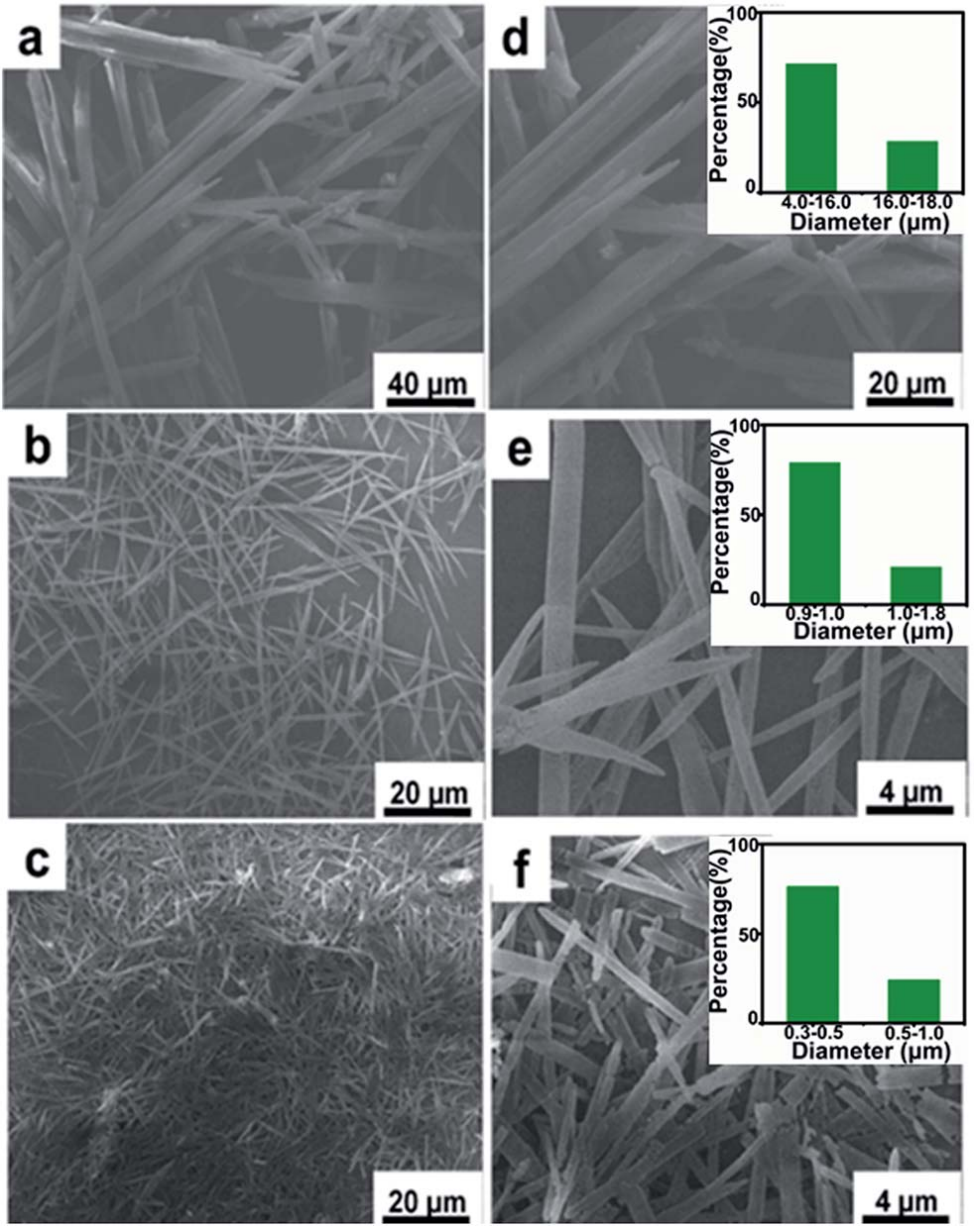
SEM images of juglone 1D nanostructures at different magnification. (a and d) Juglone micropillar, (b and e) juglone microwire, (c and f) juglone nanowire. The insert images in panels (d–f) are corresponding mathematical statistic results of juglone samples with different diameter.

Generally, the covalent bond, hydrogen bond, van der Waals forces, electrostatic forces, surface tension forces/dewetting, and π–π stacking interactions are considered as the effective factors in the self-assembly procedure of organic compounds.^34–36^ The juglone biomolecule has a α-naphthol backbone with two carbonyl groups, which may induce the molecules self-assembly by the π–π interactions.^29,37–39^ Fourier Transform Infrared Spectroscopy (FTIR) of such samples was utilized to examine the ingredient of these samples. As shown in Fig. S2,† all the samples show similar characteristic peaks, the peak at 1640 cm^−1^ is attributed to the stretching vibration of carbonyl groups, which is the main reversible redox center presented in juglone molecules. Meanwhile, Raman spectra also corroborated the results (Fig. S4†). The crystal structures of these samples are further characterized by X-ray diffraction (XRD) as shown in Fig. S3.† No impurity peak is observed in these patterns, demonstrating that these samples have the same crystal structure. And the peak intensity of juglone nanowire is stronger than juglone microwire and micropillar, suggesting that juglone nanowires have high crystallinity and relatively uniform crystal size.^30^

To investigate the redox behavior of 1D juglone micro/nanostructures, the cyclic voltammetry (CV) measurements were firstly performed (Fig. 3a), and the result reveals that these samples show a superior reversible redox performance, implying a potential application as energy storage materials. Meanwhile, compared with the CV curves of raw juglone, juglone micropillar and juglone microwire, the juglone nanowire exhibits the strongest redox peak, suggesting that the juglone nanowire-based electrode has a better charge storage behavior. To further evaluate the electrochemical performance of these samples, galvanostatic charge–discharge (GCD) measurements were carried out (Fig. 3b). First, juglone nanowire shows a higher specific capacity than that of microwire and micropillar. Besides, the plot of juglone 1D-based electrodes exhibit symmetric triangular shape with one pair of charge–discharge voltage plateau, and the voltage plateau of juglone micropillar, microwire and nanowire appears at 0.28 V, 0.26 V, 0.22 V, which is roughly consistent with the results of CV curves. Furthermore, it further reveals that the 1D nanostructure with smaller size exhibits higher specific capacity during the scan rates increasing from 10 to 200 mV s^−1^ (Fig. 3c). In detail, at a low scan rate of 10 mV s^−1^, the specific capacity delivered by juglone nanowire, juglone microwire, juglone micropillar are 389 F g^−1^, 375 F g^−1^, 116 F g^−1^, respectively, and these samples possess the specific capacities of 232 F g^−1^, 205 F g^−1^, 80 F g^−1^, when the scan rate are enhanced to 200 mV s^−1^. The general perception is that nanomaterials with higher specific surface areas usually possess better electrochemical performance.^40^ Obviously, the higher specific capacitance and superior rate capability are achieved by juglone nanowire electrode.

**Fig. 3:**
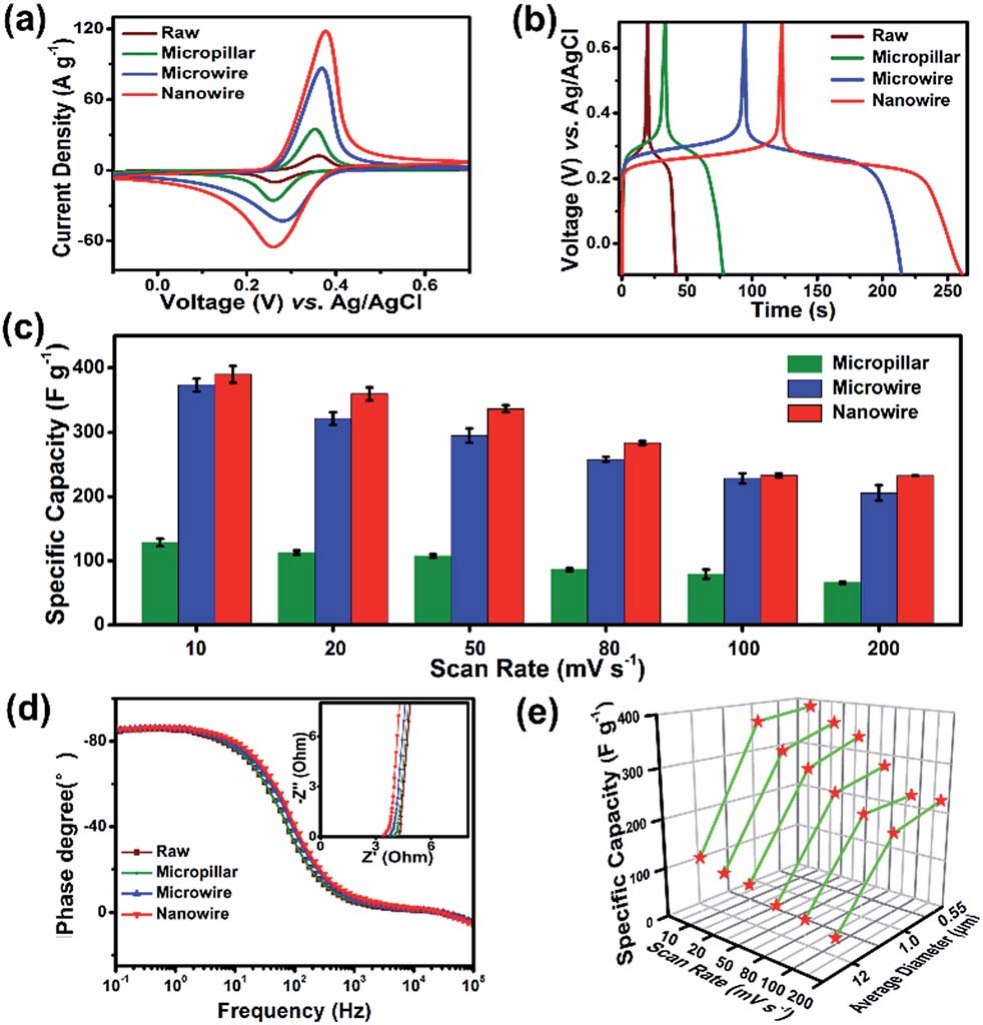
Electrochemical performance of the juglone samples with different diameter. (a) CV curves of juglone micropillar, microwire, nanowire electrodes in the potential range of −0.1 to 0.7 V (*vs.* Ag/AgCl) with a scan of 50 mV s^−1^, (b) galvanostatic charge–discharge curves of juglone electrodes with different diameter at 2 A g^−1^. (c) Statistical study of the rate performance conducted at each scan rate based on the cyclic voltammetry capacity of five electrodes as one batch. (d) Impedance phase angle as a function of frequency for juglone micropillar, microwire, nanowire electrodes. Inset is the Nyquist plot of them. (e) Correlation between the diameter and the specific capacity of the juglone samples at various scan rates.

Then, the reaction kinetics of juglone with different sizes were investigated by electrochemical impedance spectroscopy (EIS) (Fig. 3d and S5†). The crooked curve at high frequency denotes the interface resistance, involving contact and charge transfer resistances, while the low-frequency line represents ion diffusion resistance. The interface resistances of juglone nanowire, microwire, micropillar, raw material electrodes are 3.45 Ω, 3.80 Ω, 3.95 Ω, 4.02 Ω. The plot of the phase angle against the frequency reveals the characteristic frequency *f*_0_ at the phase angle of −45° is 7.46 Hz for juglone nanowire-based electrode, and it is relatively higher than that for juglone microwire (7.36 Hz), micropillar (7.25 Hz) and raw material (6.48 Hz). Accordingly, the time constant *t*_0_ (*t*_0_ = 1/*f*_0_) that represents the minimum time to discharge ≥50% of all the energy from the electrode was 0.134 s for juglone nanowire, whereas 0.136 s, 0.138 s, 0.154 s were required for juglone microwire, micropillar and raw material. The low charge transfer resistance, and short time constant validated the excellent charge and discharge capability of the juglone nanowire-based electrode.^41^

In addition, for solid-state diffusion of H^+^ in electrode materials, the mean diffusion time is proportional to the square of the diffusion path length according to the following equation:

1 *t* ≈ *L*^2^/*D*_H^+^_

where *L* is the diffusion length and *D*_H_^+^ the diffusion constant.^42,43^ It has been found that the diffusion pathway will be shortening by nanostructuring of electrode materials when the diffusion constant *D* is same,^44^ indicating that the smaller size nanostructures of juglone facilitate rapid ion/electron transport. Thus, 1D juglone nanowires possess higher specific capacitance and show slower capacity decay during the increased scan rates (Fig. 3e).

Furthermore, the CV and GCD for juglone nanowire-based electrodes were tested under various scan rates and current densities. The CV profiles show that the redox activity can be maintained very well when increasing the scan rate from 10 to 100 mV s^−1^ (Fig. 4a). Moreover, the GCD curves collected at different current densities exhibit symmetric triangular shape with one pair of charge–discharge voltage plateau, which is similar to the results of CV test (Fig. 4b and c). And the specific capacity is 342 F g^−1^ at a current density of 2 A g^−1^, it is approximately equivalent to the 345 F g^−1^ at a scan rate of 20 mV s^−1^. Next, the specific capacitance of our juglone nanowire is further compared to those of the reported conventional electrode materials.^45–51^ As shown in Fig. 4d, the capacity of N/rGO (reduced graphene oxide doping with nitrogen) is 138 F g^−1^, and our juglone nanowire have about 2-fold higher specific capacity than N/rGO, 1.5-fold higher than Fe_3_O_4_/rGO (154 F g^−1^).

**Fig. 4:**
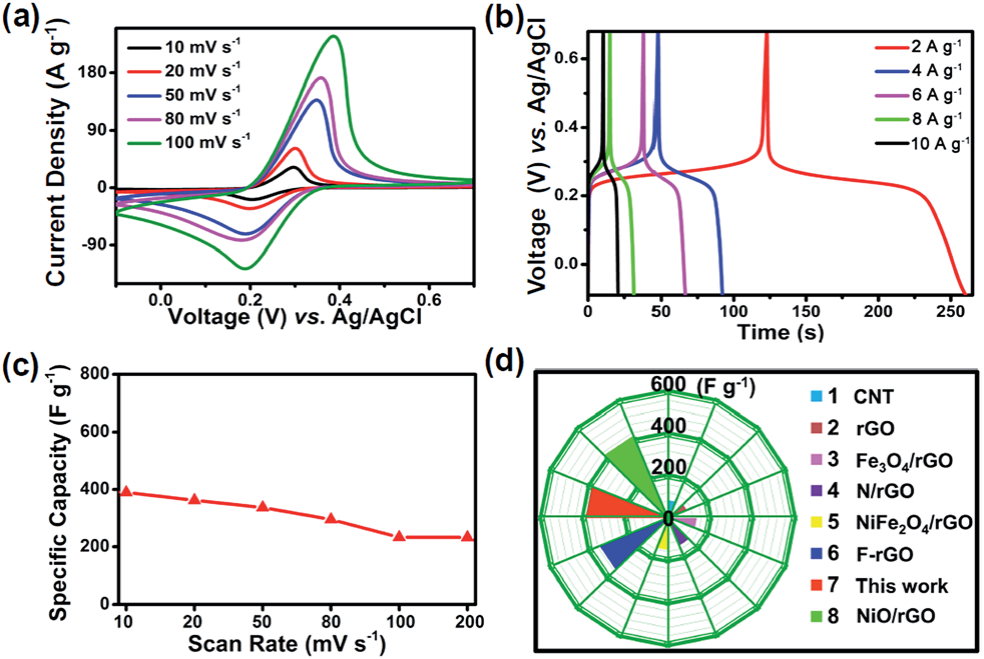
(a) CV curves of the juglone nanowire electrode at different scan rates from 10 mV s^−1^ to 100 mV s^−1^. (b) Charge and discharge curves in the current density range from 2 A g^−1^ to 10 A g^−1^. (c) Various specific capacity of the juglone nanowire electrode when increasing the scan rate from 10 mV s^−1^ to 200 mV s^−1^. (d) Specific capacity of juglone nanowire in comparison with different materials.

## Conclusions

In summary, renewable-juglone-biomolecules self-assembled 1D micro/nanostructures were successfully synthesized by an antisolvent strategy. The size effect on their charge storage properties has been investigated. It unravels that the diameter of electrode materials determines their electron/ion transport properties, the higher specific capacitance and rate capability can be achieved by the samples with the smaller diameter. The juglone nanowire delivers a remarkable high specific capacity of 389 F g^−1^ at the scan rate of 10 mV s^−1^, and retains over 68.96% of initial capacity when the scan rate up to 200 mV s^−1^. Our work reveals that the charge-storage properties of biomolecules can be modulated by the rational material-architecture design, and paves the way for the development of high-performance, environmentally-friendly and sustainable energy-storage materials.

## Conflicts of interest

There are no conflicts of interest to declare.

## Supplementary Material

RA-008-C7RA12489A-s001
