# Supplementary material for: Renewable juglone nanowires with size-dependent charge storage properties
Source: RSC Adv. 2018 Jan 9;8(4):2077–81. doi: 10.1039/c7ra12489a (PMC9077263; doi:10.1039/c7ra12489a)
Supplement: RA-008-C7RA12489A-s001 [file RA-008-C7RA12489A-s001.pdf]

Electronic Supplementary Material (ESI) for RSC Advances.

Renewable Juglone Nanowires with Size-Dependent Charge Storage Properties

Linlin Guo,[a] Aifen Wang,[b]* Pengfei Hu,[a] Aihua Tian,[a] Rui Hao,[a] Dandan Yu,[a] Jie Yang,[a] Dezhi Chen,[c]* Hua Wang[a]*

[a]School of Chemistry, Beihang University, Beijing 100191, P.R. China

[b]School of Science, Hangzhou Dianzi University, Hangzhou 310018, P.R. China

[c]Key Laboratory of Jiangxi Province for Persistent Pollutants Control and Resources Recycle, Nanchang Hangkong University, Nanchang 330063, P.R. China

1.Experimental section

Synthesis of 1D juglone samples： 40 mg, 20 mg of juglone powder (Sigma Aldrich) were dispersed in 5 mL acetonitrile, respectively. 5 mL juglone solution with a concentration of 8 mg mL^{-1} was injected into 50 mL deionized water. After 15 minutes standing, the nanowire precipitation was collected through filtration, washing and desiccation. The juglone microwire was fabricated by the same method, but the concentration of juglone solution is 4 mg mL^{-1}.

The juglone micropillar was prepared by recrystallizing a water/acetonitrile mixed solution of juglone. Specifically, 10 mg juglone was dissolved in 55 mL mixed solution of acetonitrile and deionized water (volume ratio: 1:9) with continuous stirring until homogenous solution was formed. Then, 55 mL solution was transferred to an evaporating dish and kept at room temperature for 12 h to evaporate the mixed solution.

Fabrication of 1D juglone sample electrodes： Firstly, the carbon cloth was washed with acetone, ethanol and distilled water and then dried in 70 °C for 6 h. The recrystallized juglone nanowire was mixed with carbon black and polyvinyl alcohol (PVA) binder to form slurry at the weight ratio of 2:2:1. After completely stirring, the electrode was prepared by casting the slurry onto the carbon cloth (1 cm×1 cm). The electrode was dried in a fume cupboard at room temperature overnight. The microwire, micropillar electrodes were prepared by the same process.

Materials Characterization: The morphologies of the juglone samples were characterized using a JEOL JSM-7500F cold-FESEM. The X-ray diffraction (XRD) spectra of the samples were recorded by a Rigaku Dmax 2200 X-ray diffractometer with Cu Kα radiation (λ=1.5416 Å). Raman spectroscopy measurements were performed on LabRAM HR800 system with an excitation wavelength of 633 nm. The silicon peak at 520 cm^{-1} was used as a reference. Fourier Transform Infrared Spectroscopy (FTIR) was collected using a Nicolet iN10MX instrument.

Electrochemical Measurements: Cyclic voltammogram of juglone in acetonitrile/deionized water mixed solution was captured by a three-electrode system, using a 2.3 M H_2SO_4 electrolyte and Pt foil as counter electrode and working electrode, as well as Ag/AgCl as reference electrode, with scan rates from 10 to 200 mV s^{-1}. The electrochemical performance of 1D juglone samples were performed on the CHI-660D electrochemical workstation, using a 2.3 M H_2SO_4 electrolyte, juglone electrode as work electrode, Pt foil as counter electrode, as well as Ag/AgCl as reference electrode.

2.Supporting figures

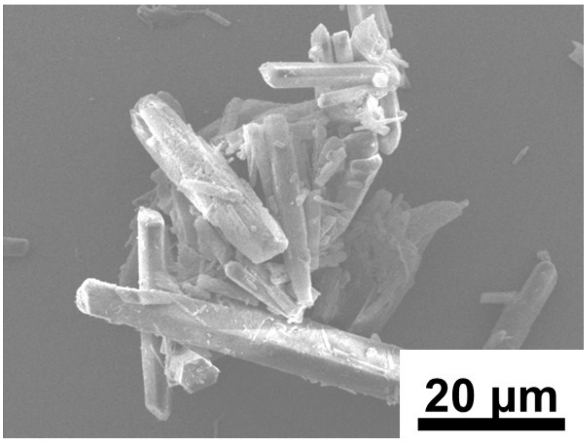

Fig S1 SEM images of raw juglone material.

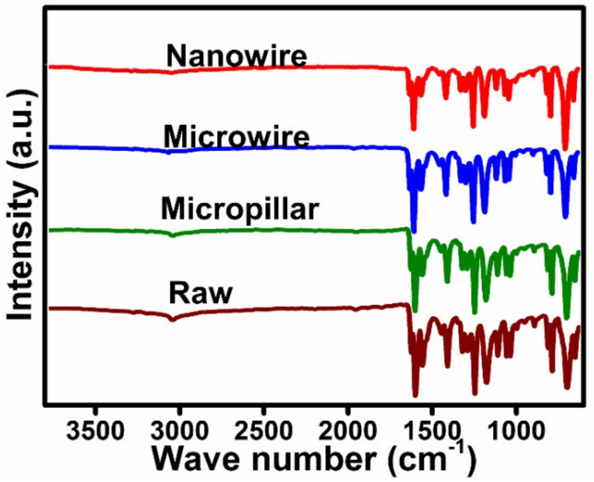

Fig S2 Fourier Transform Infrared Spectra of raw juglone and its micropillar, microwire, nanowire**.**

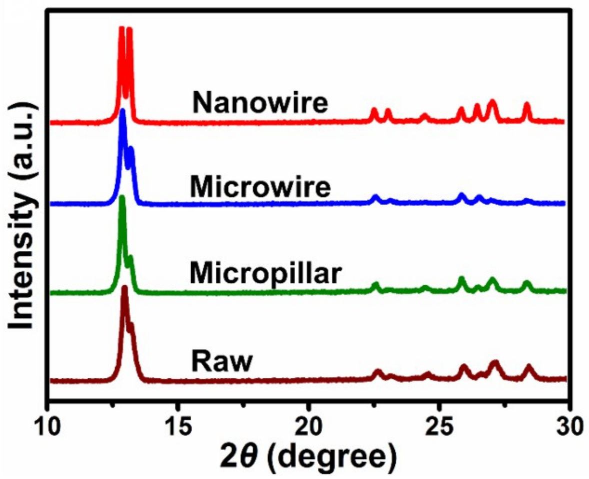

Fig S3 X-ray diffractometer patterns of raw juglone and its micropillar, microwire, nanowire**.**

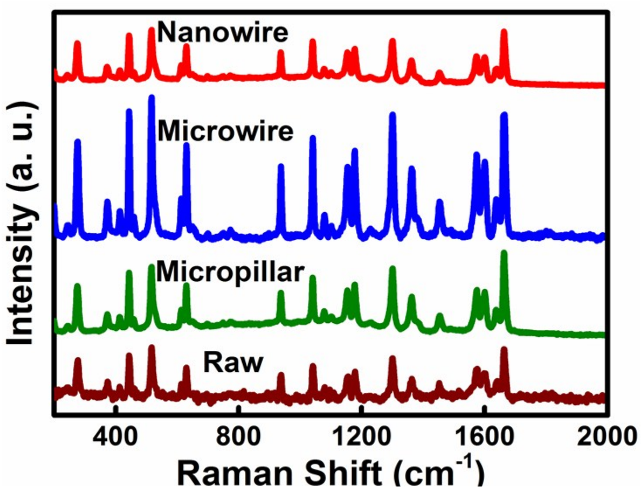

Fig S4 Raman spectra of juglone samples with different diameter.

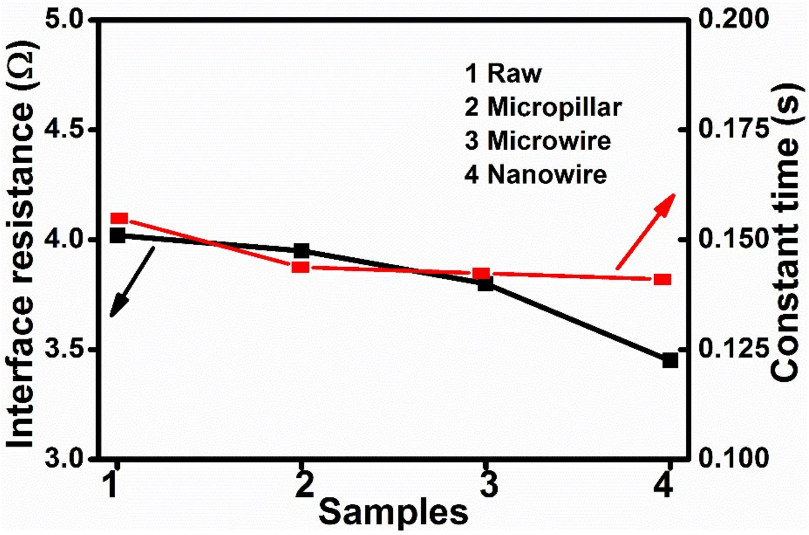

Fig S5 Linear relationship of 1D juglone samples interface resistance and constant time.
